# Prostaglandin E2 and Progesterone Receptor Coordinately Regulate Primary Cilia for Proper Decidualization

**DOI:** 10.1096/fj.202500961RR

**Published:** 2025-08-07

**Authors:** Chih‐Jhen Lee, Yung‐Chieh Tsai, Ting‐Yu Chen, Yu‐Ying Chao, Ruei‐Ci Lin, Hui‐Ling Tsai, Jen‐Yu Wen, Tang K. Tang, Pei‐Yin Tsai, Pao‐Lin Kuo, Chia‐Yih Wang

**Affiliations:** ^1^ Department of Cell Biology and Anatomy College of Medicine, National Cheng Kung University Tainan Taiwan; ^2^ Institute of Basic Medical Sciences, College of Medicine, National Cheng Kung University Tainan Taiwan; ^3^ Department of Obstetrics and Gynecology Chi‐Mei Medical Center Tainan Taiwan; ^4^ Department of Sport Management Chia Nan University of Pharmacy and Science Tainan Taiwan; ^5^ Institute of Biomedical Sciences Academia Sinica Taipei Taiwan; ^6^ Department of Biomedical Science National Chung Cheng University Chiayi Taiwan; ^7^ Department of Obstetrics and Gynecology, National Cheng Kung University Hospital, College of Medicine National Cheng Kung University Tainan Taiwan; ^8^ Department of Obstetrics and Gynecology College of Medicine, National Cheng Kung University Tainan Taiwan; ^9^ Department of Obstetrics and Gynecology E‐Da Hospital, I‐Shou University Kaohsiung Taiwan; ^10^ Department of Obstetrics and Gynecology Kaohsiung Chang Gung Memorial Hospital and Chang Gung University College of Medicine Kaohsiung Taiwan; ^11^ Department of Obstetrics and Gynecology Jen‐Ai Hospital Taichung Taiwan

**Keywords:** cAMP, decidualization, primary cilium, progesterone receptor, prostaglandin E2

## Abstract

Decidualization, the process by which endometrial stromal cells differentiate into decidual cells in response to prostaglandin E2 (PGE2) and progesterone receptor (PGR) signaling, is essential for proper implantation and placentation. Primary cilia, microtubule‐based cellular antennae, contribute to various differentiation processes, including decidualization. In this study, we demonstrated that both the proportion of ciliated cells and ciliary length increased in a time‐dependent manner during in vitro decidualization. In a mouse model, after copulation, the proportion of ciliated cells fluctuated, but ciliary length progressively increased over time. Additionally, we observed defective primary cilia in the endometrium of women with recurrent miscarriages. Mechanistically, we found that primary cilia were present before the expression of decidual markers under decidual stimulation. Depletion or inhibition of primary cilia impaired decidualization, highlighting their critical role in this process. Furthermore, we identified the PGE2–PKA–CREB1 axis as a key regulator of ciliary growth and PGR upregulation. Upon progesterone stimulation, active PGR further increased ciliary length, thereby facilitating decidualization. Thus, our study not only establishes a link between ciliary length and decidualization but also elucidates the sequential regulation of ciliary dynamics by PGE2 and PGR in a coordinated manner.

## Graphical abstract

Prostaglandin E2 (PGE2) activates the PKA‐CREB1 pathway to promote primary ciliogenesis and upregulates progesterone receptor (PGR) expression in endometrial stromal cells. Progesterone binds to PGR, enhancing the elongation of primary cilia. These processes drive the differentiation of stromal cells into decidual stromal cells, initiating and maintaining decidualization. PGE2 and progesterone synergistically and sequentially regulate primary cilia, which are essential for successful decidualization.

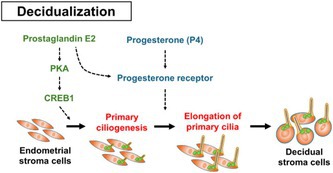

## 1 Introduction

Primary cilia play a crucial role in cell differentiation and have multiple functions in female reproduction. Unlike motile cilia, primary cilia are single, immotile cellular protrusions that function as sensory organelles. They integrate extracellular signals and transmit them into the cell to regulate differentiation [1, 2]. Disruption of primary cilia impairs differentiation and has been linked to various diseases [3, 4]. Structurally, primary cilia consist of a central axoneme, intraflagellar transporter (IFT) machinery, and a surrounding ciliary membrane. The axoneme, composed of microtubules extending from the mother centriole, serves as the ciliary backbone. The IFTs facilitate bidirectional protein transport to facilitate and maintain primary cilia. The ciliary membrane encases the axoneme and harbors signaling receptors that receive environmental cues and transduce them into the cell to regulate cellular physiology [5]. Defective primary cilia are associated with pregnancy complications, including gestational diabetes mellitus (GDM) and preeclampsia [6, 7]. In early pregnancy, primary cilia are present on trophoblast cells, where they play a crucial role in mediating signaling pathways, including those activated by endocrine gland‐derived vascular endothelial growth factor (EG‐VEGF) [7, 8]. EG‐VEGF signals through receptors localized on the primary cilia, highlighting their importance in this pathway. By facilitating EG‐VEGF signaling, primary cilia support proper trophoblast invasion, a process essential for successful placental development.

The formation of primary cilia, known as primary ciliogenesis, is a tightly regulated process. Under normal conditions, both the mother and daughter centrioles are capped proteins that suppress cilia formation. When ciliogenesis is initiated, tau tubulin kinase 2 (TTBK2) is recruited to the mother centriole, where it phosphorylates CP110 and CEP83, leading to their removal from the mother centriole [9, 10]. This event exposes the mother centriole, allowing the docking of ciliary vesicles, a crucial step for ciliogenesis. Following vesicle docking, the axoneme extends outward, accompanied by the recruitment of IFT protein complexes, which facilitate axoneme elongation and ciliary growth [11]. The progressive extension of the axoneme ultimately gives rise to the fully formed primary cilium, enabling its role as a signaling hub [5].

During pregnancy, the uterine endometrium undergoes dramatic molecular and morphological changes for embryo implantation and development; this process is now known as decidualization [12, 13]. When decidualization occurs, the elongated fibroblast‐like endometrial stromal cells (ESCs) differentiate into a rounded and specialized secretory epithelioid cell type, termed decidual stromal cells (DSCs) [14]. DSCs accumulate glycogen and lipid droplets in the cytoplasm and upregulate the abundance of secretory proteins including prolactin (PRL) and insulin‐like growth factor binding protein‐1 (IGFBP‐1), two established markers of decidualization [15]. After decidualization, uterine spiral arteries begin to remodel and increase in diameter. Concurrently, macrophages, large granular lymphocytes, and uterine natural killer cells infiltrate the stromal compartment to regulate endovascular trophoblast invasion and support the formation of a functional feto‐maternal interface that is receptive to the embryo [16, 17]. Thus, disturbances in the decidualization process cause severe pregnancy complications such as implantation failure and pregnancy loss, infertility, recurrent miscarriages, and intrauterine fetal growth restriction [18].

Prostaglandin E2 (PGE2) and the progesterone receptor (PGR) are key regulators of decidualization, orchestrating stromal cell differentiation, immune adaptation, and vascular remodeling in the endometrium [19, 20]. PGE2 facilitates decidualization by activating the cyclic adenosine monophosphate (cAMP) pathway, which enhances the expression of essential decidualization markers such as insulin‐like growth factor‐binding protein 1 (IGFBP1) and prolactin [21, 22]. Additionally, upregulation of PGR expression further facilitates stromal cell responsiveness to progesterone, thereby promoting decidualization [23]. The interplay between PGE2 and PGR ensures a well‐coordinated decidualization process, which is essential for implantation and the maintenance of early pregnancy [24].

Progesterone, acting through PGR, is one of the most critical regulators of decidualization. PGR belongs to the nuclear receptor superfamily (NR3C3) and exists in two major isoforms, PGR‐A and PGR‐B, both of which regulate overlapping and distinct gene sets crucial for decidualization and pregnancy establishment [25]. Studies in knockout models have demonstrated that the absence of both PGR isoforms leads to infertility due to defects in embryo implantation and uterine decidualization [26]. Interestingly, PGR‐A knockout female mice exhibit phenotypes similar to those of double‐knockout mice, suggesting that PGR‐A is the predominant mediator of decidualization [27]. Notably, PGR localizes to motile cilia and facilitates ciliary beating in ciliated epithelial cells of the fallopian tubes in both mice and humans [28], further highlighting its diverse functional roles beyond traditional genomic regulation.

This study demonstrated that the proportion of ciliated cells and ciliary length increased during in vitro decidualization. In addition, we observed defective primary cilia in the endometrium of women with recurrent miscarriages. During decidualization, primary cilia were present before the expression of decidual markers. Depletion or inhibition of primary cilia impaired decidualization, highlighting their critical role in this process. Furthermore, we identified that the PGE2‐PKA‐CREB1 axis initiated the growth of primary cilia. Upon progesterone stimulation, PGR was activated and increased the ciliary length, thereby facilitating decidualization. Thus, our study not only establishes a link between the length of primary cilia and decidualization but also elucidates the sequential regulation of ciliary dynamics by PGE2 and PGR in a coordinated manner.

## 2 Materials and Methods

### 2.1 Cell Cultures

The telomerase‐immortalized human endometrial stromal (T‐HESC) cell line and human endometrial stroma primary culture cells were maintained in Roswell Park Memorial Institute (RPMI)‐1640 medium supplemented with 10% charcoal‐stripped serum, 2 g/L sodium bicarbonate, 1% sodium pyruvate, 1% penicillin/streptomycin, and 1% glutamine. The cells were incubated in a humidified atmosphere containing 95% air and 5% CO_2_ at 37°C. Mycoplasma contamination was regularly examined using immunofluorescence staining and immunoblotting assays.

### 2.2 In Vitro Decidualization

Decidualization was induced in vitro by treating cells for three days with 10 μM medroxyprogesterone 17‐acetate (MPA; Sigma, #1378001, St. Louis, USA) and 0.3 mM 8‐bromo‐cAMP (Enzo, #BML‐CN115, NY, USA). Prostaglandin E2 (PGE2) induces decidualization by increasing the levels of cAMP [29]. Alternatively, decidualization was also induced using 10 μM MPA combined with 1 μM PGE2 (Sigma, #P0409, St. Louis, USA).

### 2.3 Detection of IGFBP1 and Prolactin

The supernatants from different treatments were loaded into the protein‐coated 96‐well ELISA plates, including IGFBP1 (ab233618, Abcam, Cambridge, UK) and prolactin (ab226901, Abcam, Cambridge, UK). The antibody cocktail, consisting of the capture antibody, detector antibody, and diluent, was prepared. Subsequently, 50 μL of supernatant and 50 μL of the antibody cocktail were added to each well, followed by shaking for one hour at 400 rpm. The wells were aspirated and washed three times with wash buffer. TMB was then added for color development. Once the color had developed, the reaction was stopped using stop solution, and the optical density (OD) was measured at 450 nm.

### 2.4 Flow Cytometry

Cells were detached using trypsin, collected by centrifugation at 500 × *g*, and washed three times with PBS. After the final wash, the supernatant was removed, and the cell pellet was resuspended in PBS and fixed with ice‐cold 70% ethanol for 24 h. After fixation, cells were washed three times with PBS and then incubated with 100 μg/mL RNase A and 40 μg/mL propidium iodide (PI) for 30 min at room temperature. DNA content was analyzed using a FACSCalibur flow cytometer (BD Biosciences).

### 2.5 EdU Incorporation Assay

To examine the cell proliferation, proliferating cells were labeled by using the Click‐iT EdU Cell Proliferation Kit (C10337; Invitrogen; Waltham; USA). Cells were seeded on coverslips and incubated with EdU for one hour. The cells were then fixed with cold methanol and washed three times with PBS, followed by shaking for 15 min. Next, the cells were incubated with Click‐iT buffer, Alexa Fluor azide, and CuSO_4_ for one hour at room temperature in the dark. The nuclei were stained with DAPI. The cells were then washed three times with PBS, shaken for 30 min, and the coverslips were mounted. Proliferating cells were analyzed using an Axio Imager M2 fluorescence microscope (Zeiss, Oberkochen, Germany).

### 2.6 Western Blotting

The treated cells were lysed in lysis buffer with protease inhibitor, and the lysates were centrifuged at 12000 rpm for 12 min to get the supernatant, and the supernatant was collected. When the supernatant was collected, it was quantified by Bradford protein assay. The quantified lysates were mixed with the 2X sample buffer and boiled at 110°C for 10 min. Samples were resolved by SDS‐PAGE and transferred onto PVDF membranes, which were washed with TBST for 30 min on the shaker, then blocked with 3% BSA/TBST for one hour. Following blocking, the membranes were incubated with primary antibodies overnight at 4°C. Next, the primary antibodies were washed out with TBST for 30 min, followed by adding the secondary antibodies and reacting for one hour. After washing, the protein signals were detected using an ECL kit (WBKLS0500; Millipore; Burlington; USA).

### 2.7 Immune‐Fluorescence Microscopy

Cells were seeded on glass coverslips and allowed to attach for one day prior to drug treatment. Following the treatment, cells were then fixed with cold methanol. Blocking was performed using PBS buffer containing Triton X‐100, Tween‐20 (PBST), and normal goat serum, and incubated for one hour at room temperature. Primary antibodies were then applied and incubated overnight at 4°C. The following day, coverslips were washed three times with PBST and incubated for one hour with DAPI for nuclear staining, along with secondary antibodies for detecting specific cellular structures. After staining, coverslips were washed three additional times with PBST, protected from light, and placed on a shaker for 30 min. Finally, the coverslips were mounted with antifade mounting medium, and stained cells were imaged using an Axio Imager M2 fluorescence microscope (Zeiss, Oberkochen, Germany).

### 2.8 Animal Models

C57BL/6 mice were purchased from National Cheng Kung University Laboratory Animal Center (Tainan; Taiwan). For the natural pregnancy decidualization model, fertile female mice were mated with fertile male mice. The day the vaginal plug was observed was defined as gestation Day 1. The samples of mouse endometrium stroma from Day 1 to 4 were collected separately, and the primary cilia were measured. All animal procedures were approved by the Institutional Animal Care and Use Committee (IACUC) of National Cheng Kung University, in accordance with institutional guidelines and national regulations. The approval number for this study is IACUC:112187.

### 2.9 Human Tissue Samples

The study was conducted using endometrial tissue samples stored in the Chi Mei Medical Center Biobank, and all eligible participants had previously signed informed consent forms for participation in the Human Biobank, agreeing to provide tissue samples for future academic research.

Endometrial tissue samples were collected via endometrial sampling with a Pipelle from women in the proliferative phase. The menstrual cycle phase was determined based on the dates of the previous cycle. The tissue samples were fixed in formalin and subsequently embedded in paraffin for future sectioning.

The study included women aged over 20 years. Women older than 40 years, with a BMI ≥ 30, a history of smoking, previous miscarriage with confirmed chromosomal abnormalities, or a history of preeclampsia, eclampsia, gestational diabetes, pregnancy‐induced hypertension, or chronic hypertension were excluded. Recurrent pregnancy loss was defined as two or more consecutive miscarriages before a gestational age of 20 weeks. Tissue samples from multiparous women without a history of recurrent pregnancy loss served as the control group. Human sample analysis was approved by the Institutional Review Board of the Chi Mei Medical Center: 11012‐004.

### 2.10 Reagents

20 μM of Roscovitine (R7772; Sigma; St. Louis; USA), 10 μM H89 (HY‐15979A; MCE; NJ; USA), 100 μM Mifepristone (475 838; Sigma; St. Louis; USA).

### 2.11 RNA Interference

siRNA transfections were performed in T‐HESC cells to silence the expression of IFT88, CEP164, and the progesterone receptor (PGR). IFT88 and CEP164 are essential regulators of primary cilia formation and function and were targeted to investigate the role of ciliary signaling in decidualization.

For siRNA transfection, 500 μL of Opti‐MEM was mixed with 6 μL of Lipofectamine 2000 (Invitrogen). Separately, siRNA oligonucleotides were diluted in Lipofectamine 2000 and incubated for 5 min. The two solutions were then combined and allowed to incubate at room temperature for 30 min to allow complex formation. T‐HESC cells were cultured in 1 mL of RPMI‐1640 medium and transfected with 1 mL of the siRNA‐Lipofectamine mixture. Cells were incubated under standard culture conditions for 72 h.

The following siRNA oligonucleotides were purchased from Dharmacon Scramble control siRNA (siCTL): 5′‐ugguuuaacaugucgacuaa[dt][dt]‐3′.

To disrupt primary cilia, the following siRNA was used:

- siIFT88: 5′‐cgacuaagugccagacucauu[dt][dt]‐3′.
- siCEP164: 5′‐caggugacauuuacuauuuca[dt][dt]‐3′.

To disrupt progesterone receptor, the following siRNA was used:

- siPGR#1: 5′‐gcuacgaagucaaacccaguu[dt][dt]‐3′.
- siPGR#2: 5′‐caauacagcuucgagucauua[dt][dt]‐3′.

shRNA transfections were conducted in T‐HESE cells and purchased from the RNAi core lab of Genomics Research Center of Academia Sinica (Taipei; Taiwan). In the shRNA transfection experiment, T‐HESC cells were infected with lentivirus containing shRNA.

shRNA against luciferase (shLuc) is used as a scramble control: 5′‐cguacgcggaaauacuucga[dt][dt]‐3′.

To disrupt primary cilia, the following shRNA was used:

- shCREB1#1: 5′‐acagcacccacuagcacuauu[dt][dt]‐3′.
- shCREB1#2: 5′‐acggugccaacuccaauuuac[dt][dt]‐3′.
- shCREB1#3: 5′‐gcuagaggaaaguggagauuu[dt][dt]‐3′.
- shCREB1#4: 5′‐cggaggaagauucgaaauaaa[dt][dt]‐3′.

### 2.12 Antibodies

For analyzing the cell cycle, the following antibodies were used: anti‐cyclin E (#4132, Cell Signaling, Beverly, MA, USA). For examining decidualization, the following antibodies were used: anti‐CREB1 (#9197, Cell Signaling), anti‐PGR (GTX22765, GeneTex, Irvine, USA), anti‐PGR (phospho‐Ser190; GTX30164, GeneTex), anti‐TTBK2 (HPA018113, Sigma, St. Louis; USA), anti‐CREB3 (ab180119, Abcam, Cambridge, UK) and anti‐HAND2 (ab200040, Abcam). For examining primary cilia, the following antibodies were used: anti‐acetylated tubulin (T6793, Sigma), anti‐CP110 (ab99338, Abcam), anti‐ARL13b (17711–1‐AP, Proteintech, Chicago, USA), anti‐IFT88 (13967‐1‐AP, Proteintech), and anti‐CEP164 (NBP1‐81445, Novus, CO, USA). The following antibodies are used as internal controls: anti‐ku70 (#4588, Cell Signaling), anti‐beta tubulin (GTX101279, GeneTex), and anti‐GAPDH (CAB0004, Cyrusbioscience, Taipei, Taiwan).

### 2.13 Statistical Analysis

All quantitative results were presented as means ± standard deviation (SD), obtained from at least three independent experiments. More than 300 cells were counted in each individual group. Statistical significance was determined using unpaired two‐tailed *t*‐tests for pairwise comparisons and one‐way ANOVA for multiple group comparisons. *p*‐values less than 0.05 were considered statistically significant.

## 3 Results

### 3.1 Endometrial Stromal Cells Develop Primary Cilia During Decidualization

Successful differentiation of uterine endometrial stromal cells, a process known as decidualization, is essential for proper implantation [30]. Primary cilia have been observed during decidualization, and defective primary ciliogenesis has been reported in the endometrium of patients with recurrent miscarriages, highlighting the importance of primary cilia in this process [31, 32]. However, the regulation of primary cilia during decidualization remains largely unexplored.

**FIGURE 1:**
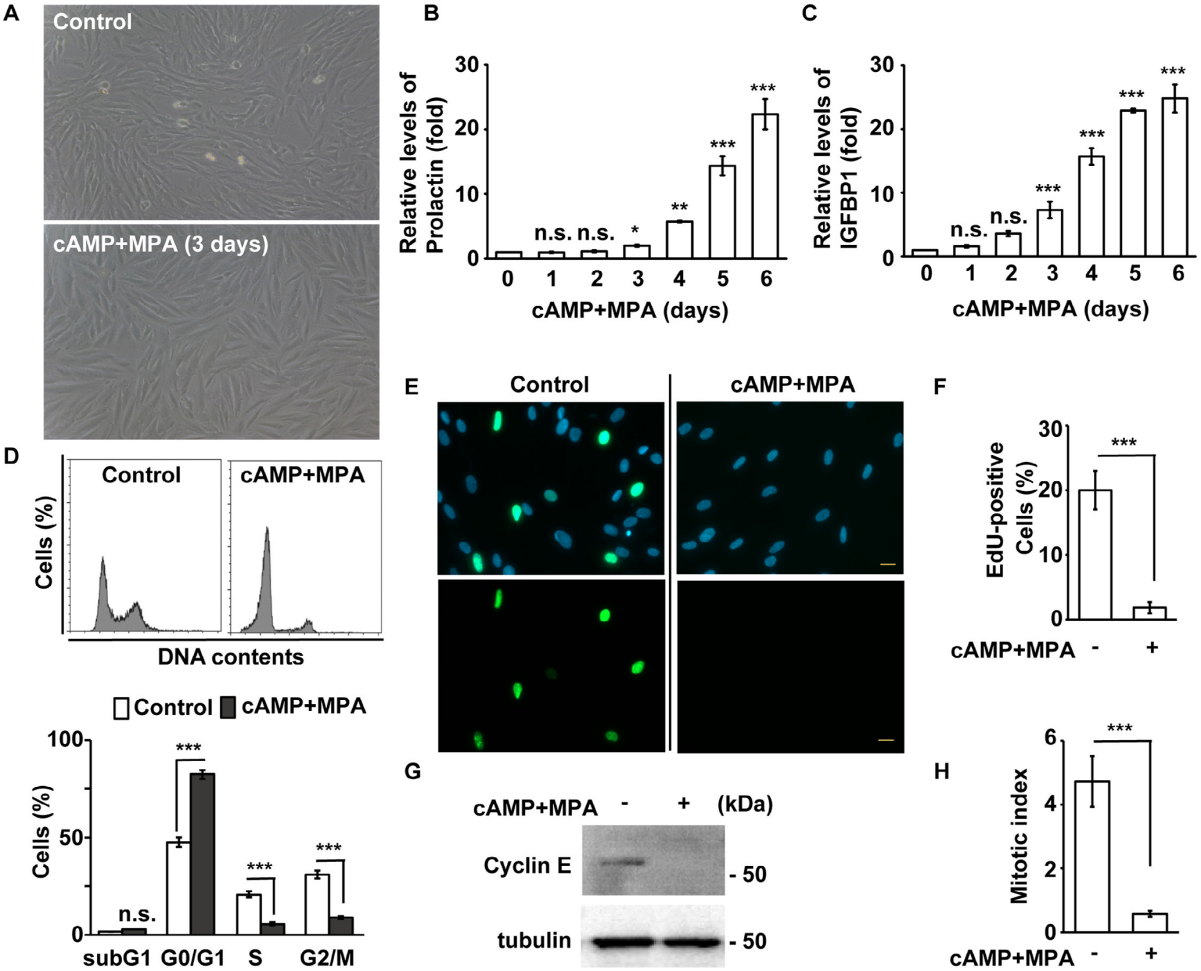
Decidualization induces cell cycle arrest in the G0/G1 phase. (A) The morphology changed in the absence (upper panel, control) or presence (lower panel, cAMP+MPA) of decidual stimulation in T‐HESC cells as shown by light microscopy. Scale bar, 40 μm. (B, C) Increased expressions of decidual markers, Prolactin (B) and IGFBP1 (C), in a time‐dependent manner. (D–H) Decidualization induces cell cycle arrest in the G0/G1 phase. (D) Cell cycle profiles were analyzed using flow cytometry. (E, F) Reduced S phase entry as shown by EdU assay. (E) Lower EdU‐positive cells were observed in cells treated with cAMP and MPA. (F) Quantitative result of (E). (G) The expression of cyclin E was reduced during decidualization. Cells were treated with MPA and cAMP, followed by Western blotting using antibodies against cyclin E and tubulin. (H) The proportion of mitotic cells (mitotic index) was reduced upon cAMP and MPA treatment. n.s.: no significance, *p < 0.05, **p < 0.01, ***p < 0.001. These results are mean ± SD from three independent experiments. At least 300 cells were counted in each individual group.

To investigate the underlying molecular mechanisms, an in vitro decidualization assay was performed. Treatment with cAMP and medroxyprogesterone acetate (MPA, a progesterone analog) induced decidualization [33]. Upon decidual stimulation, achieved by treating immortalized human endometrial stromal cells (T‐HESCs) with cAMP and MPA for three days, the spindle‐shaped T‐HESCs adopted a rounded, secretory decidual morphology, indicating successful decidualization (Figure 1A). To further confirm this, the expression of decidual markers, including prolactin and IGFBP1, was examined. Both prolactin and IGFBP1 levels were significantly upregulated after decidual stimulation in a time‐dependent manner (Figure 1B,C). These findings demonstrate that decidualization was successfully induced in vitro.

During differentiation, the cell cycle was arrested in the G0/G1 phase [34]. Since decidualization is a differentiation process, cell cycle profiles were analyzed using flow cytometry. Upon decidual stimulation, decidualized cells were arrested in the G0/G1 phase, with a reduced proportion of cells in the S and G2/M phases (Figure 1D). This was further confirmed by a lower number of EdU‐positive cells, downregulation of cyclin E, and a reduced mitotic index (Figure 1E–H). Our data suggest that T‐HESCs are arrested in the G0/G1 phase during decidualization.

**FIGURE 2:**
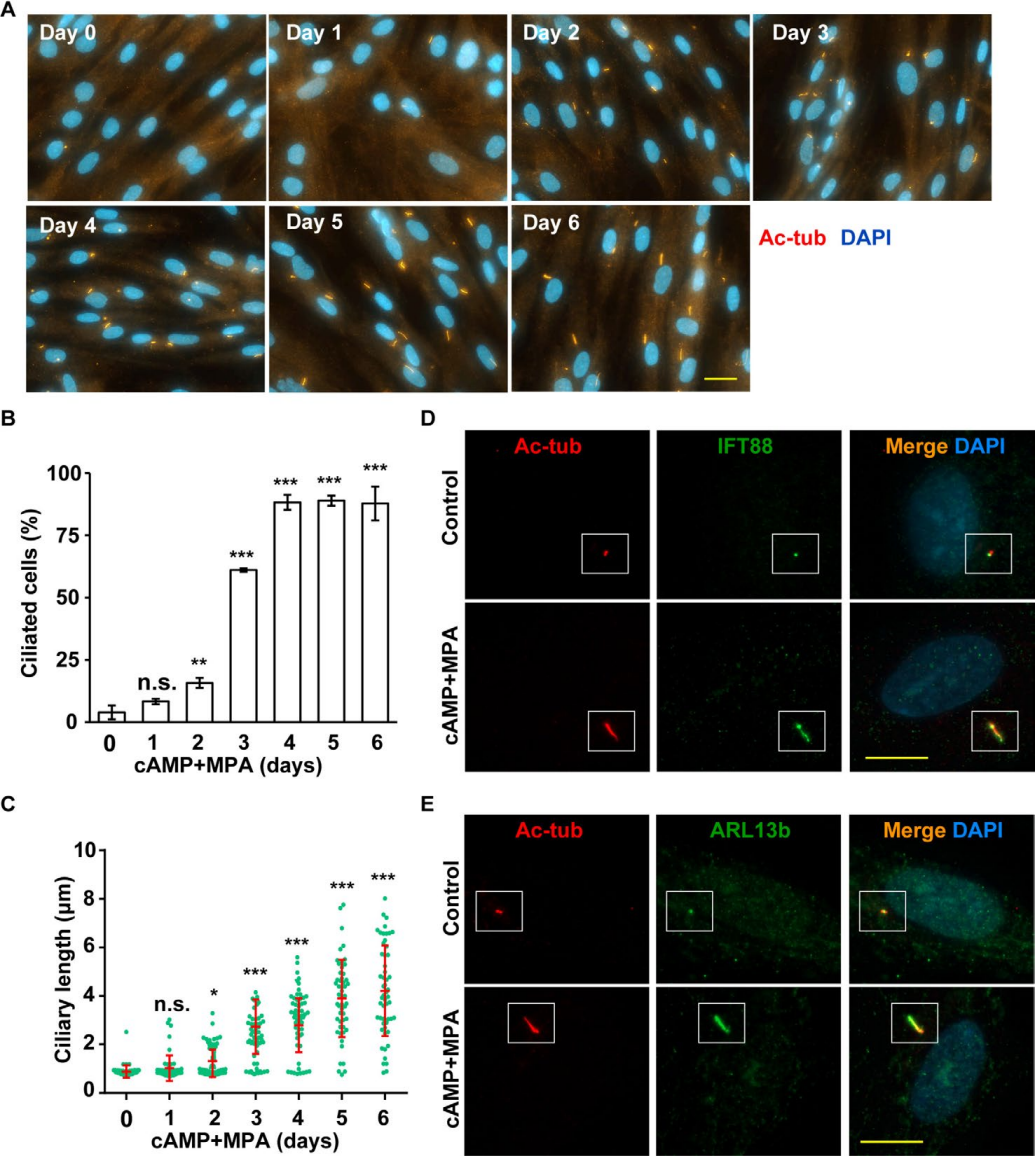
Decidual stimulation induces primary cilia formation in T‐HESC cells. (A–C) Co‐treatment of cAMP and MPA promoted primary ciliogenesis in a time‐dependent manner. (A) Primary ciliogenesis was induced by decidual stimulation. Primary cilia were observed by immunofluorescence staining with antibodies against acetylated tubulin (Ac‐tub, red). DNA were stained with DAPI (blue). Scale bar, 20 μm. (B) Quantitative result of the proportion of ciliated cells upon co‐treatment of cAMP and MPA. (C) Quantitative result of the length of primary cilia upon co‐treatment of cAMP and MPA. (D, E) Ciliary structures were examined by immunofluorescence staining. (D) Primary cilia were examined by immunofluorescence staining with antibodies against acetylated tubulin (Ac‐tub, D and E), IFT88 (D) and ARL13b (E). DNA were stained with DAPI (blue). Scale bar, 10 μm. n.s. no significance, *p < 0.05, **p < 0.01, ***p < 0.001. These results are mean ± SD from three independent experiments. At least 300 cells were counted in each individual group.

Cells typically develop primary cilia during the G0/G1 phase, and primary cilia are known to promote cell differentiation [35]. Therefore, we examined primary ciliogenesis during decidualization. Two days after decidual stimulation, T‐HESCs began forming primary cilia, with both the proportion of ciliated cells and the length of primary cilia increasing in a time‐dependent manner (Figure 2A–C). Immunofluorescence staining was then performed to further analyze these cilia. Primary cilia‐related markers, including axonemes (as indicated by acetylated tubulin, Figure 2D,E), intraflagellar transport components (as shown by IFT88 staining, Figure 2D), and ciliary membranes (as indicated by ARL13b staining, Figure 2E), were observed, suggesting that these cilia contained essential ciliary components.

Next, we investigated the initiation of primary ciliogenesis. At the onset of ciliogenesis, TTBK2 is recruited to the mother centriole, leading to the removal of CP110 [10]. When T‐HESCs underwent decidualization, TTBK2 was recruited to the base of primary cilia, while CP110 was removed (Figure S1A,B). These findings suggest that decidual signals induce primary ciliogenesis via a canonical pathway.

**FIGURE 3:**
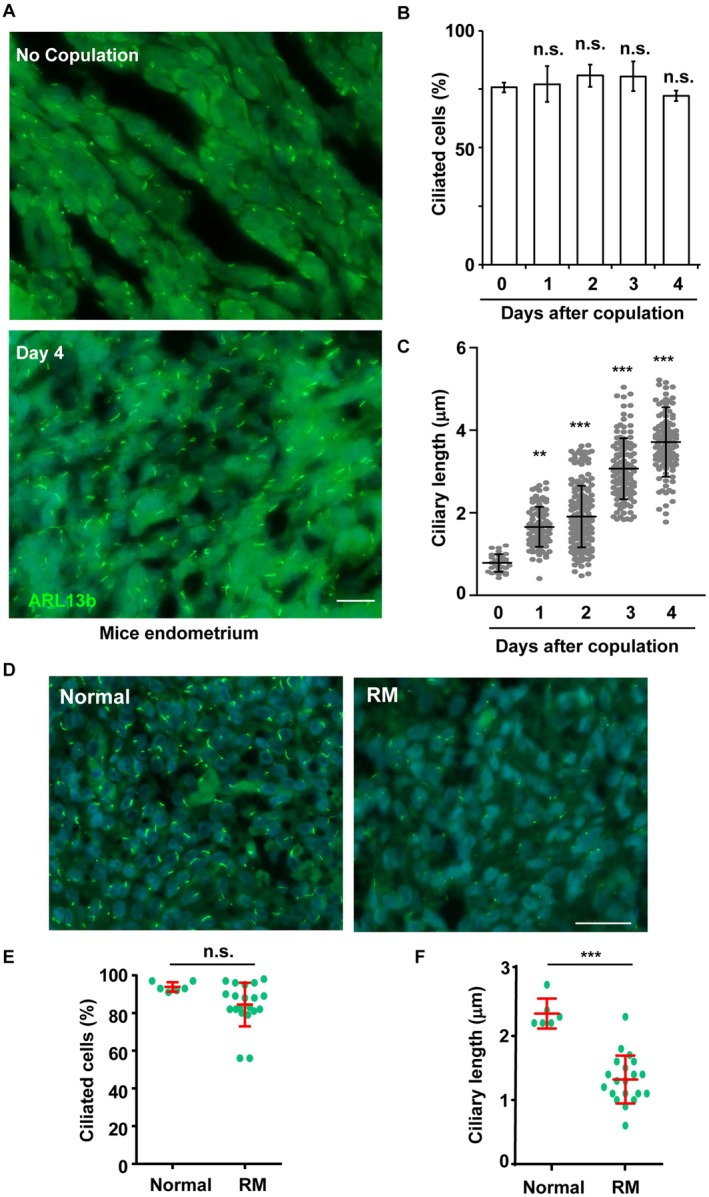
Endometrial stroma grows primary cilia in vivo. (A–C) The length of primary cilia increased after mice copulation. (A) Primary cilia in the endometrial stroma after copulation were observed by immunofluorescence staining with an antibody against ARL13b (green). DNA were stained with DAPI. Scale bar, 20 μm. (B) Quantitative result of the proportion of ciliated cells in (A). (C) Quantitative result of the length of primary cilia in (A). (D–F) Defective primary cilia were shown in women with recurrent miscarriages. (D) Primary cilia in normal proliferative phase endometrium (*N* = 6) or the endometrium of women with recurrent miscarriages (*N* = 20) were observed by immunofluorescence staining with an antibody against ARL13b (green). DNA were stained with DAPI. Scale bar, 20 μm. (E) Quantitative result of the proportion of ciliated cells in (D). (F) Quantitative result of the length of primary cilia in (D). n.s.: no significance, *p < 0.05, **p < 0.01, ***p < 0.001. These results are mean ± SD from three independent experiments. At least 300 cells were counted in each individual group.

To further validate our observations, we examined primary cilia formation in the uterine endometrium of mice following copulation. In the absence of copulation, primary cilia were present in the uterine endometrial cells (Figure 3A). Notably, 4 days after copulation, during the period of active decidualization, the proportion of ciliated cells remained unchanged; however, the length of primary cilia increased in a time‐dependent manner (Figure 3B,C). Additionally, we examined primary cilia in human endometrial samples (Figure 3D). Immunofluorescence staining was performed to assess primary cilia in endometrial tissues from control women and patients with recurrent miscarriages, both during the proliferative phase. The percentage of ciliated cells was comparable between the two groups, indicating no significant difference in the proportion of ciliogenesis (Figure 3E). However, the average length of primary cilia was significantly reduced in the recurrent miscarriages group compared to controls (Figure 3F), suggesting impaired ciliary elongation may be associated with defective endometrial function in recurrent miscarriages. Although these observations were made during the proliferative phase, which precedes decidualization, they provide insight into the basal status of ciliogenesis in the endometrium prior to differentiation. The in vivo mouse model, in contrast, captures the dynamic regulation of cilia length during active decidualization, thus complementing this temporal limitation. These findings suggest that primary cilia play a critical role during early pregnancy.

### 3.2 Primary Cilia Contribute to Endometrial Decidualization

During the in vitro decidualization assay, T‐HESC cells developed primary cilia 2 days after decidual stimulation, whereas decidual markers were detected three days after stimulation. This suggests that primary cilia formation occurs prior to decidualization. We therefore hypothesized that primary cilia formation contributes to decidualization.

**FIGURE 4:**
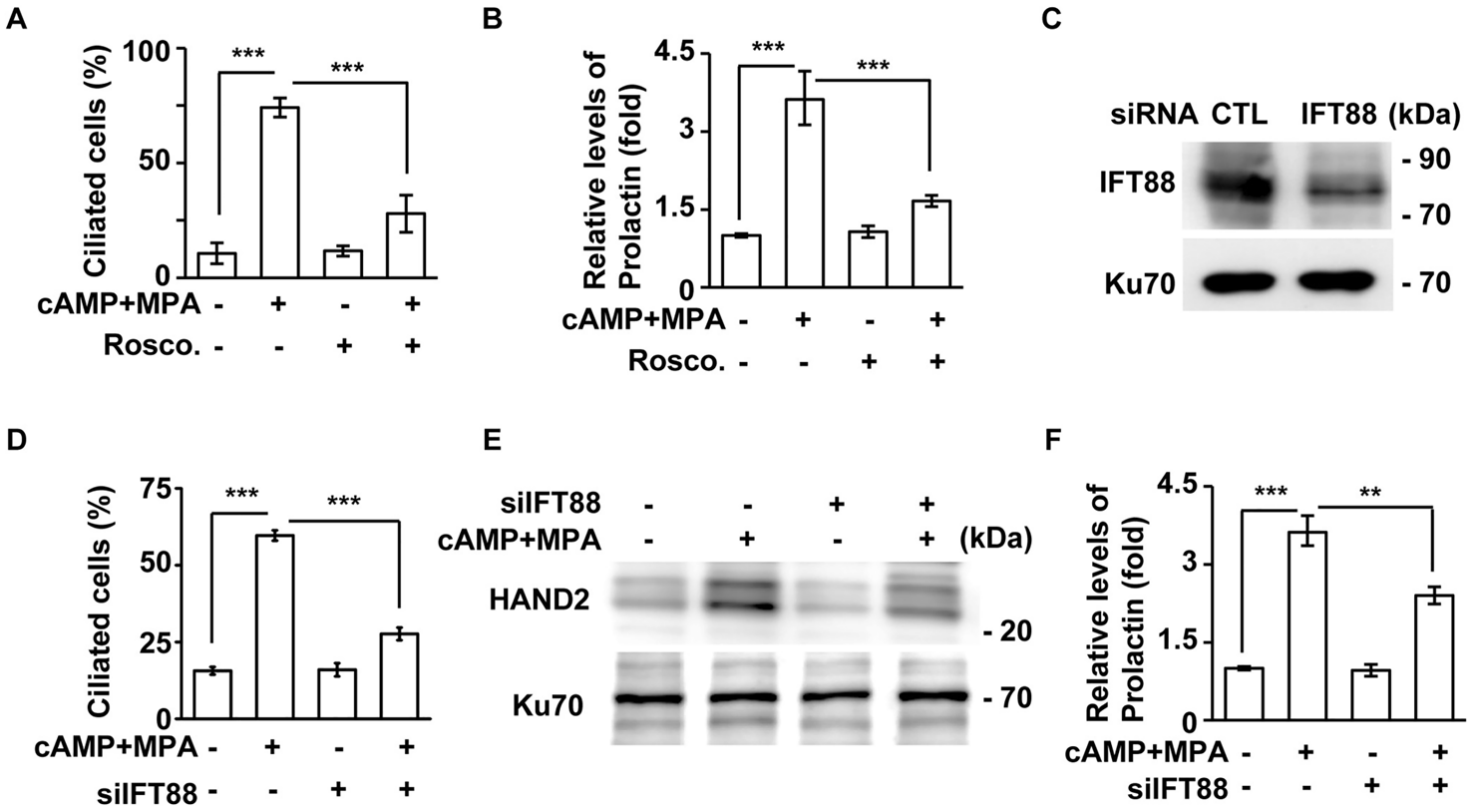
Disruption of primary cilia inhibits decidualization. (A, B) Inhibition of primary cilia by treating cells with roscovitine (Rosco.) reduced primary cilia and decidualization. (A) Quantitative results of the proportion of ciliated cells in the absence or presence of decidual stimulation (cAMP+MPA) or roscovitine. (B) The expression of the decidual marker Prolactin was reduced in roscovitine‐treated cells. (C–F) Depletion of ciliary gene IFT88 reduced decidualization. (C) IFT88 was successfully depleted by transfecting T‐HESC cells with siRNA against IFT88. Extracts of siRNA‐transfected cells were analyzed by Western blotting with antibodies against IFT88 and Ku70 (loading control). (D) The proportion of ciliated cells were reduced in IFT88‐deficient cells. (E, F) Depletion of primary cilia impaired decidualization. (E) The expressions of decidual marker HAND2 (E) and Prolactin (F) were reduced in IFT88‐deficient T‐HESC cells. **p < 0.01, ***p < 0.001. These results are mean ± SD from three independent experiments. At least 300 cells were counted in each individual group.

To test this hypothesis, we first examined whether blocking primary cilia inhibits decidualization. When cells were treated with roscovitine, a known inhibitor of primary cilia formation [36, 37], the proportion of ciliated cells was significantly reduced (Figure 4A), and the expression of decidual markers, prolactin and IGFBP1, was also significantly decreased (Figure 4B; Figure S2A). These experiments were also conducted in primary endometrial stromal cells derived from clinical samples (primary‐cultured cells; Figure S2B–D). However, as roscovitine is a broad‐spectrum cyclin‐dependent kinase inhibitor that lacks specificity, we further employed a genetic approach to validate our observations. We depleted the ciliary gene IFT88 by transfecting cells with siRNA against IFT88 (Figure 4C). Depletion of IFT88 inhibited primary cilia formation (Figure 4D) and reduced the expression of decidual markers HAND2 and prolactin (Figure 4E,F). These phenotypes were also observed when another ciliary gene, CEP164, was depleted (Figure S2E–G). Taken together, our data suggest that primary cilia are required for proper decidualization.

**FIGURE 5:**
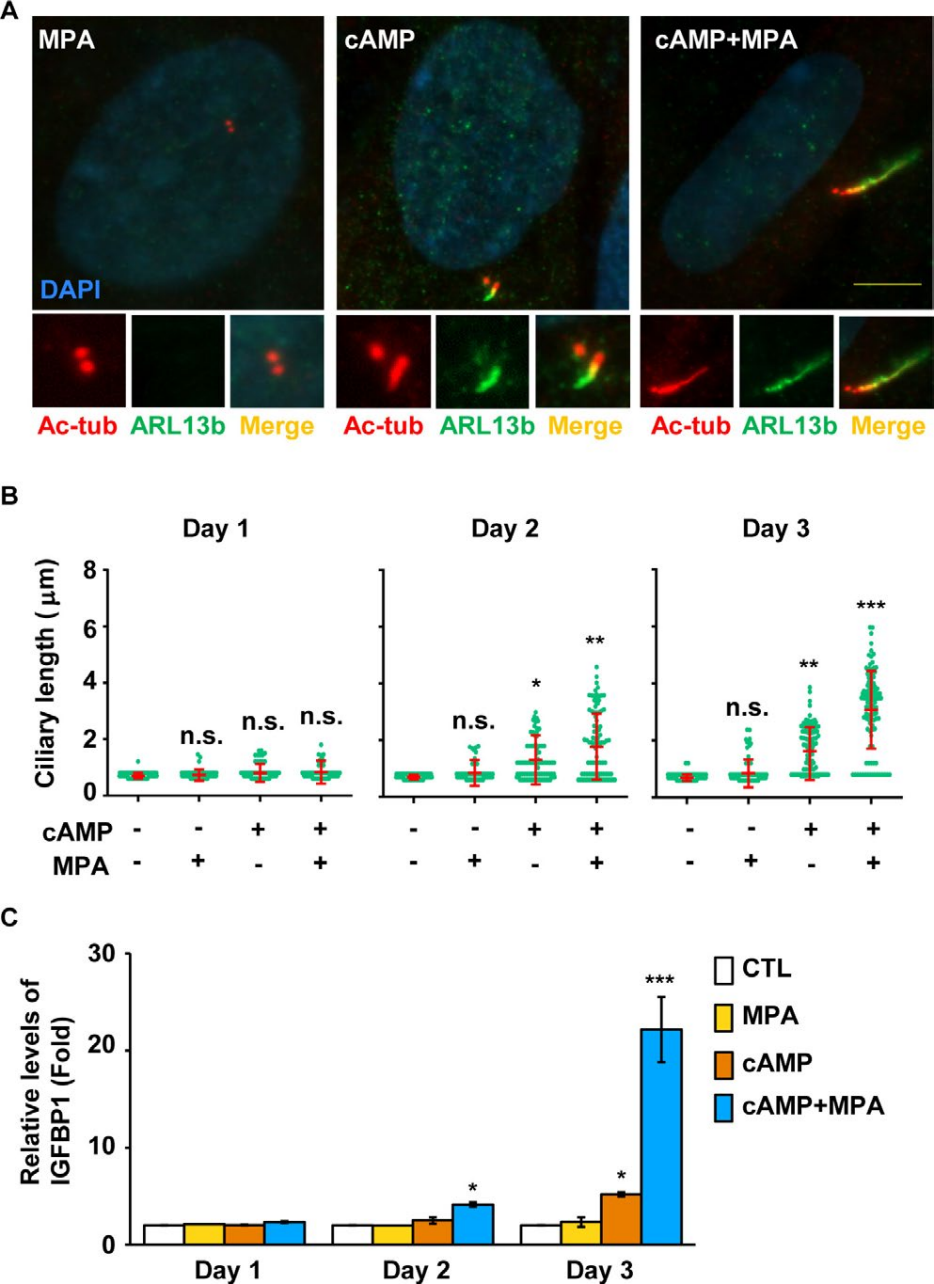
The ciliary length was coordinately regulated by cAMP and MPA. (A–C) MPA treatment did not induce primary cilia, whereas cAMP induced primary cilia growth. Co‐treatment with cAMP and MPA resulted in longer primary cilia. (A) Primary cilia were shown by immunofluorescence staining with antibodies against acetylated tubulin (Ac‐tub, red) and ARL13b (green) in MAP‐, cAMP‐, or cAMP+MPA‐treated T‐HESC cells. DNA were stained with DAPI (blue). Scale bar, 10 μm. (B) Quantitative result of the length of primary cilia of cells in the absence or presence of cAMP or MPA for one, two, or three days. (C) Quantitative result of decidual marker IGFBP1 in the absence or presence of cAMP or MPA for one, two, or three days. CTL: control. n.s.: no significance, *p < 0.05, **p < 0.01, ***p < 0.001. These results are mean ± SD from three independent experiments. At least 300 cells were counted in each individual group.

Since decidualization is triggered by co‐treating cells with cAMP and MPA, we next investigated how these signaling molecules contribute to primary ciliogenesis. Treatment with MPA alone had no effect on primary ciliogenesis (Figure 5A, left panel). However, cAMP induced primary ciliogenesis two days after treatment, a time point at which decidual markers were not yet expressed (Figure 5A, middle panel; Figure 5B, Day 2; Figure 5C, Day 2). The length of primary cilia increased three days after cAMP treatment, at which point IGFBP1 expression was also induced. Co‐treatment with cAMP and MPA resulted in longer primary cilia as early as 2 days after treatment, with further elongation observed three days after treatment. Additionally, IGFBP1 expression was significantly higher compared to cells treated with cAMP alone (Figure 5C, Day 3). Thus, our findings suggest that cAMP induces primary ciliogenesis, while MPA enhances ciliary elongation, thereby facilitating decidualization.

### 3.3 PGE2 Induces Primary Ciliogenesis, and Progesterone Promotes Cilia Elongation During Decidualization

**FIGURE 6:**
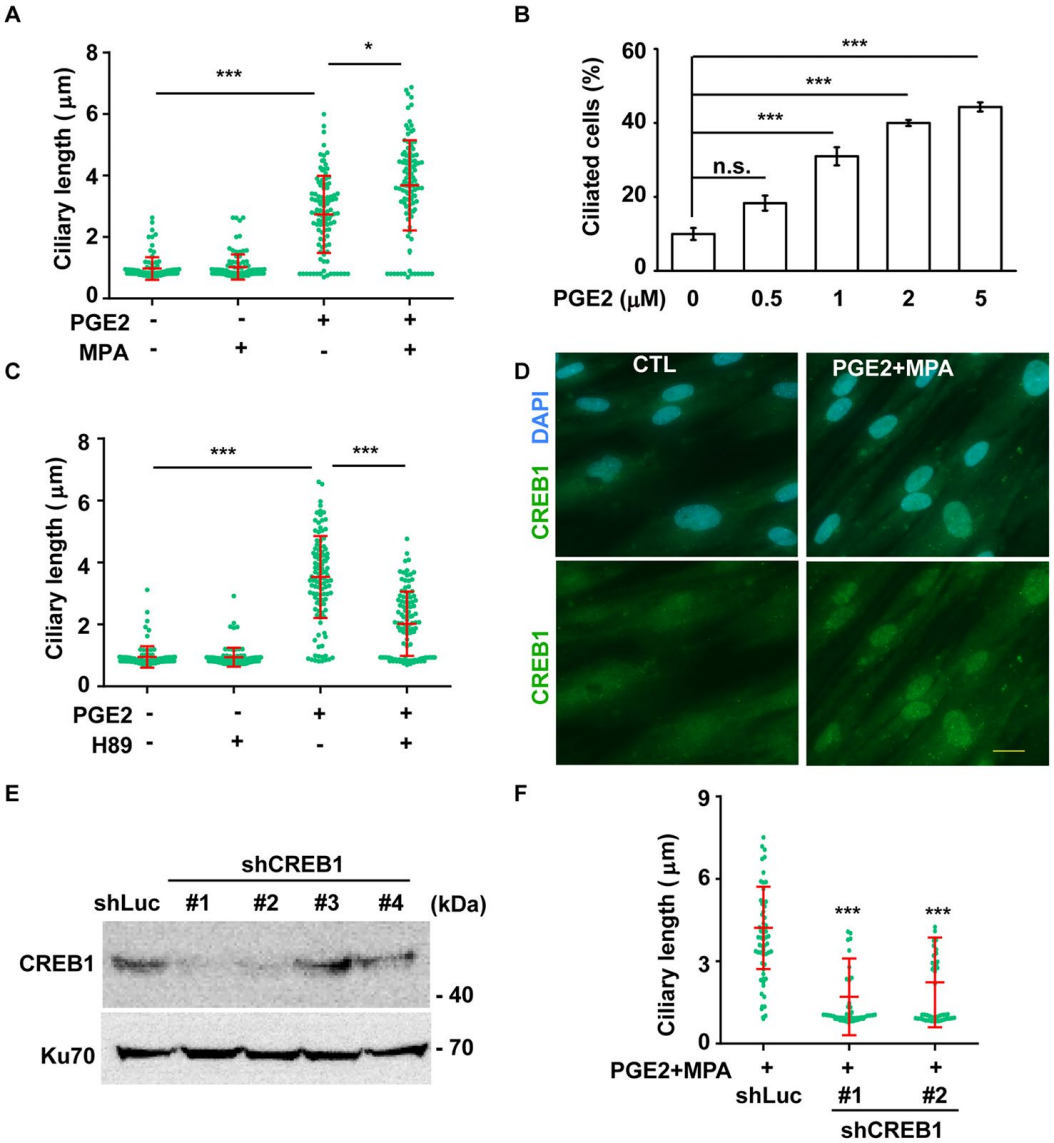
PGE2 promotes primary ciliogenesis to facilitate decidualization. (A) PGE2 promoted primary cilia formation. Quantitative result of the length of primary cilia when cells were treated with PGE2, MPA, and PGE2 + MPA. (B) PGE2 promoted primary ciliogenesis in a dose‐dependent manner. Quantitative result of the proportion of ciliated cells in the presence of PGE2 at different doses. (C) Inhibition of PKA by treating cells with H89 reduced the length of primary cilia. Quantitative result of the length of primary cilia in the absence or presence of PGE2 or H89. (D) Co‐treating cells with PGE2 and MPA activated CREB1. Nuclear localization of CREB1 was detected by immunofluorescence staining with antibodies against CREB1 (green). DNA were stained with DAPI (blue). Scale bar, 20 μm. (E, F) CREB1 induced primary cilia. (E) Depletion of CREB1 by infecting cells with lentivirus containing shRNA against CREB1 (shCREB1#1, #2, #3, and #4). Extracts of CREB1 deficient cells were analyzed by Western blotting with antibodies against CREB1 and Ku70. shLuc: shRNA against luciferase, negative control. (F) Depletion of CREB1 reduced primary cilia. Quantitative result of ciliary length of control or CREB1‐deficient cells. n.s.: no significance, *p < 0.05, ***p < 0.001. These results are mean ± SD from three independent experiments. At least 300 cells were counted in each individual group.

PGE2 activates PKA by upregulating cAMP to induce decidualization [38]. Since treatment with cAMP was sufficient to induce primary ciliogenesis, we next tested whether PGE2 could also induce primary ciliogenesis. Similar to cAMP, treatment with PGE2 induced the formation of primary cilia, and co‐treatment with MPA further enhanced ciliary length (Figure 6A). Besides, the effect of PGE2 on primary cilia was dose‐dependent (Figure 6B). Next, we examined whether PGE2 induced primary cilia via PKA activation. While PGE2 induced primary ciliogenesis, co‐treatment with the PKA‐selective inhibitor H89 reduced primary cilia formation (Figure 6C), suggesting that PGE2‐induced ciliogenesis occurs through PKA activation.

PKA activates CREB, leading to its phosphorylation and nuclear translocation, thereby triggering decidualization [39, 40]. Our data showed that the PGE2‐PKA axis induced primary ciliogenesis, prompting us to investigate whether CREB was involved in this process. Upon co‐treatment with PGE2 and MPA, CREB1, but not CREB3, was upregulated and translocated into the nucleus (Figure 6D; Figure S3A), suggesting that CREB1 activation was associated with PGE2‐induced decidualization. To determine the role of CREB1 in promoting primary ciliogenesis, we depleted CREB1 using lentiviral transduction with four distinct shRNA sequences targeting CREB1 (shCREB1 #1, #2, #3, and #4). Three days after lentiviral infection, shCREB1 #1 and #2 efficiently reduced CREB1 expression (Figure 6E). Furthermore, CREB1 depletion resulted in reduced ciliary length following PGE2 and MPA co‐treatment (Figure 6F). Collectively, our findings suggest that the PGE2‐PKA‐CREB1 axis induces primary ciliogenesis, and ciliary length is further enhanced in the presence of progesterone.

### 3.4 Progesterone Receptor (PGR) Activation Contributes to Primary Ciliogenesis

**FIGURE 7:**
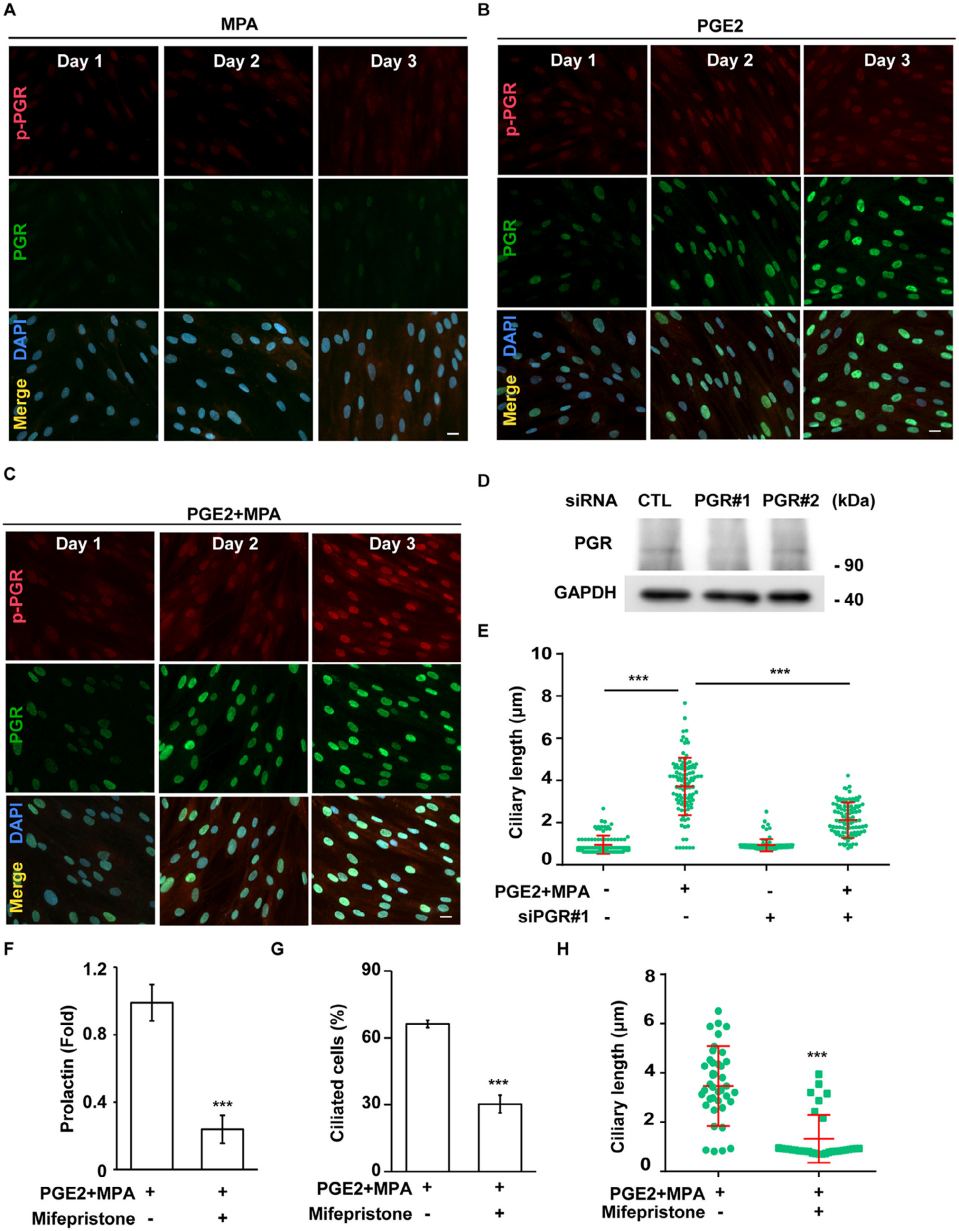
Activation of PGR promotes the elongation of primary cilia during decidualization. (A) Treatment of MPA alone had no effect on PGR. (B) Treatment of cAMP induced, but not activated, the expression of PGR. (C) Co‐treatment of cAMP and MPA induced and activated the PGR. (A–C) Active PGR (phosphorylated PGR, p‐PGR) and PGR were examined by immunofluorescence staining with antibodies against p‐PGR (red) and PGR (green). DNA were stained with DAPI (blue). Scale bar, 20 μm. (D, E) Depletion of PGR reduced the primary cilia upon decidualization. (D) The expression of PGR was successfully depleted. Extracts of PGR‐deficient (T‐HESC cells were transfected with two siRNA against PGR: siPGR#1 and siPGR#2) were analyzed by Western blotting with antibodies against PGR and GAPDH. (E) Depletion of PGR reduced the length of primary cilia. Quantitative result of the length of primary cilia. (F–H) Inhibition of PGR by treating cells with mifepristone reduced primary cilia and decidualization. (F) Quantitative result of the expression of decidual marker prolactin under decidualization. (G) Quantitative result of the proportion of ciliated cells with or without mifepristone under decidualization. (H) Quantitative result of the length of primary cilia with or without mifepristone under decidualization. ***p < 0.001. These results are mean ± SD from three independent experiments. At least 300 cells were counted in each individual group.

Activation of the progesterone receptor (PGR) is essential for decidualization; however, its role in primary ciliogenesis remains poorly understood. Since co‐treatment with PGE2 and MPA induced primary ciliogenesis, we investigated their effects on PGR expression and activation. Treatment with MPA alone neither upregulated nor activated PGR (Figure 7A). Interestingly, treatment with PGE2 alone for 2 days increased PGR expression, with further upregulation observed three days after PGE2 treatment (Figure 7B). Despite this increase in PGR expression, PGR was not activated. However, in T‐HESC cells co‐treated with PGE2 and MPA, PGR expression increased in a time‐dependent manner, and PGR activation was confirmed by the presence of phosphorylated PGR in the nucleus (Figure 7C). These findings suggest that while PGE2 upregulates PGR expression, MPA is required for its activation.

To assess whether PGR activation contributes to primary ciliogenesis, we inhibited PGR expression using siRNA (siPGR #1 and #2). siPGR #1 effectively suppressed PGR expression (Figure 7D), and PGR depletion inhibited the ciliary elongation induced by PGE2 and MPA co‐treatment (Figure 7E), suggesting that PGR plays a role in primary ciliogenesis. To further validate this observation, we blocked PGR activity using its selective inhibitor, mifepristone. Mifepristone treatment inhibited prolactin expression, reduced the proportion of ciliated cells, and shortened primary cilia length (Figure 7F–H). Thus, our findings indicate that PGE2 upregulates PGR expression, while MPA activates PGR to promote primary ciliogenesis.

### 3.5 Primary Cilia Maintain Decidualization

**FIGURE 8:**
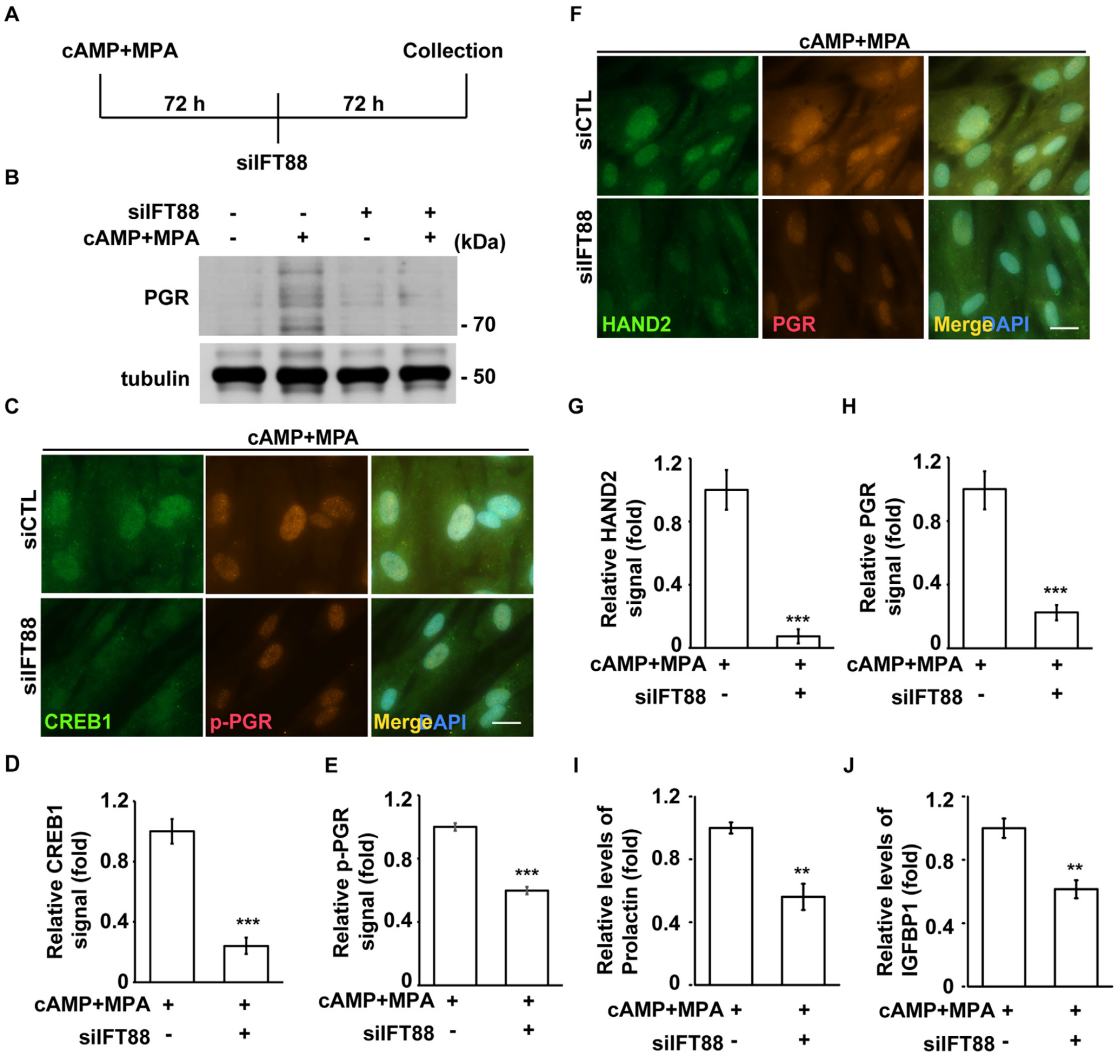
Primary cilia maintain decidualization. (A) The schematic model of experimental designs. (B–J) Depletion of IFT88 impairs decidualization. (B) Western blot analysis showing reduced expression of progesterone receptor (PGR) in cells transfected with siRNA targeting IFT88 (siIFT88), with or without cAMP+MPA treatment. Tubulin was used as a loading control. (C) Immunofluorescence staining of CREB1 and phosphorylated PGR (p‐PGR) in control and siIFT88‐transfected cells treated with cAMP+MPA. Nuclei were counterstained with DAPI (blue). Scale bar, 20 μm. (D, E) Quantification of fluorescence intensity for (D) CREB1 and (E) p‐PGR, measured using ImageJ. (F) Immunofluorescence staining of HAND2 and PGR under the same conditions as (C). Scale bar, 20 μm. (G, H) Quantification of fluorescence intensity for (G) HAND2 and (H) PGR. (I, J) ELISA analysis of (I) prolactin and (J) IGFBP1 secretion following IFT88 depletion. Quantitative data are presented as mean ± SD from three independent experiments. At least 300 cells were analyzed per condition for immunofluorescence quantification. Fluorescence intensities were measured using ImageJ. **p < 0.01, ***p < 0.001.

Our data suggest that decidual signaling induces primary ciliogenesis, which in turn promotes decidualization. We next investigated whether primary cilia are required to maintain decidualization. To test this, we first induced decidualization for three days and then disrupted primary cilia by transfecting cells with siRNA targeting IFT88 for an additional three days under decidualization conditions (Figure 8A). IFT88 depletion reduced PGR expression, suggesting that disruption of primary cilia impairs the maintenance of decidualization (Figure 8B). This finding was further supported by immunofluorescence staining, which showed reduced nuclear expression of CREB1, phosphorylated PGR, total PGR, and HAND2 in primary cilia‐deficient cells (Figure 8C–H). Additionally, ELISA analysis revealed decreased levels of prolactin and IGFBP1 upon IFT88 depletion (Figure 8I,J). Collectively, these results suggest that decidual signaling induces primary ciliogenesis, which in turn is essential for maintaining decidualization.

## 4 Discussion

Primary cilia play a crucial role in development and differentiation. Previous studies have shown that primary cilia are present in the endometrial stroma [41]. Moreover, defective primary cilia have been observed in the endometrium of women with recurrent miscarriages, suggesting a potential link between primary cilia and successful pregnancy. Prostaglandin E2 (PGE2) and progesterone‐induced endometrial decidualization are essential for embryo implantation. In this study, we demonstrated that primary cilia begin to form prior to decidualization and that disruption of primary cilia impairs decidualization, even when the process is initiated. This finding suggests that primary cilia not only induce decidualization but also play a role in maintaining this differentiation process. Additionally, we found that PGE2 stimulates the formation of primary cilia and upregulates PGR expression. Upon progesterone stimulation, PGR is activated, further promoting the elongation of primary cilia, which is required for decidualization (Figure S4). Thus, our study underscores the critical role of primary cilia in both facilitating and maintaining decidualization and elucidates the sequential regulation of primary cilia by PGE2 and progesterone.

Our findings demonstrate that progesterone promotes primary ciliogenesis through PGR. However, the precise role of PGR‐mediated primary ciliogenesis in decidualization remains unclear. Indian Hedgehog (IHH), a member of the Hedgehog signaling family primarily associated with bone development, is also expressed in the uterine luminal epithelium during early pregnancy [42]. Notably, IHH expression in the endometrium is regulated by progesterone [43]. When progesterone binds to stromal PGR, it induces IHH expression in the epithelium, initiating a signaling cascade that activates Smoothened (SMO) and nuclear receptor subfamily 2 group F member 2 (NR2F2) in stromal cells. This activation influences the cell cycle by preventing progression into the M phase. During the transition from the early to the mid‐secretory phase of the menstrual cycle, IHH expression declines significantly, coinciding with reduced cell division [44]. As T‐HESCs differentiate into decidual stromal cells, cell proliferation ceases, supporting the notion that IHH modulates the cell cycle to maintain endometrial receptivity and facilitate embryo implantation. Disruption of IHH signaling leads to cell cycle dysregulation, ultimately resulting in decidualization failure [42]. Based on these observations, we propose that progesterone/PGR signaling induces T‐HESC cells to enter a quiescent phase, during which primary cilia formation is initiated.

The activation of ciliogenesis has been shown to induce autophagy, and conversely, autophagy plays a regulatory role in ciliogenesis [45]. Specifically, during cilia‐induced autophagy, the pre‐autophagosomal marker autophagy‐related 16‐like 1 (ATG16L1) is transported in vesicles containing IFT20 to the base of the primary cilia [46]. At this location, autophagosome formation may occur either at the plasma membrane or within the ciliary pocket, facilitating the degradation of OFD1, a negative regulator of ciliogenesis [47]. In addition to its role in ciliogenesis, autophagy has recently been implicated in decidualization. Studies have shown that ATG16L1 deficiency leads to decidualization failure in T‐HESC cells, suggesting that autophagy is essential for this process [48]. Given the interplay between ciliogenesis and autophagy, we propose that autophagy not only regulates ciliogenesis but also serves as a critical mediator through which primary cilia influence decidualization. However, further studies are required to elucidate the precise mechanisms underlying this relationship.

Brain and Muscle ARNT‐Like 1 (BMAL1) is a key regulator of the circadian clock in humans and has recently been implicated in decidualization. Studies have shown that BMAL1 dysregulation impairs the ability of decidual stromal cells to regulate trophoblast invasion, leading to implantation defects and recurrent miscarriage [49]. The differentiation of T‐HESC cells into DSCs is a critical step in decidualization, and abnormal differentiation has been linked to pregnancy failure and miscarriage. Interestingly, BMAL1 knockout mice exhibit defective motile cilia in the pulmonary airway epithelium, suggesting a potential role for BMAL1 in ciliogenesis [50]. Given these findings, it is plausible that BMAL1 may contribute to decidualization not only through its canonical circadian function but also by regulating primary cilia formation. However, further studies are required to validate this hypothesis and elucidate the underlying mechanisms.

In this study, we have discovered that primary cilia contribute to decidualization. Upon decidual stimulation, PGE2 induces the growth of primary cilia and upregulates the expression of PGR. Subsequently, upon progesterone stimulation, PGR is further activated, leading to the elongation of primary cilia, which helps maintain decidualization.

## Author Contributions

C‐.J.L. and C‐.Y.W. designed research. C‐.J.L., T‐.Y.C., Y‐.Y.C., R‐.C.L., and H‐.L.T. performed research. Y‐.C. T., J‐.Y.W., P‐.Y.T., and P‐.L.K. contributed new reagents or tissue samples. C‐.J.L., H‐.L.T., T.K.T., P‐.Y.T., P‐.L.K., and C‐.Y.W. analyzed data. C‐.J.L. and C‐.Y.W. wrote the paper.

## Conflicts of Interest

The authors declare no conflicts of interest.

## Supporting information


**Figures S1–S4:** fsb270919‐sup‐0001‐FiguresS1‐S4.docx.

## Data Availability

Data sharing not applicable to this article as no datasets were generated or analyzed during the current study.
