# Supplementary material for: Prostaglandin E2 and Progesterone Receptor Coordinately Regulate Primary Cilia for Proper Decidualization
Source: FASEB J. 2025 Aug 7;39(15):e70919. doi: 10.1096/fj.202500961RR (PMC12329624; doi:10.1096/fj.202500961RR)
Supplement: Supplementary file 1 — Figures S1–S4: fsb270919‐sup‐0001‐FiguresS1‐S4.docx. [file FSB2-39-e70919-s001.docx]

**Supplemental information**

Supplementary Figure 1.


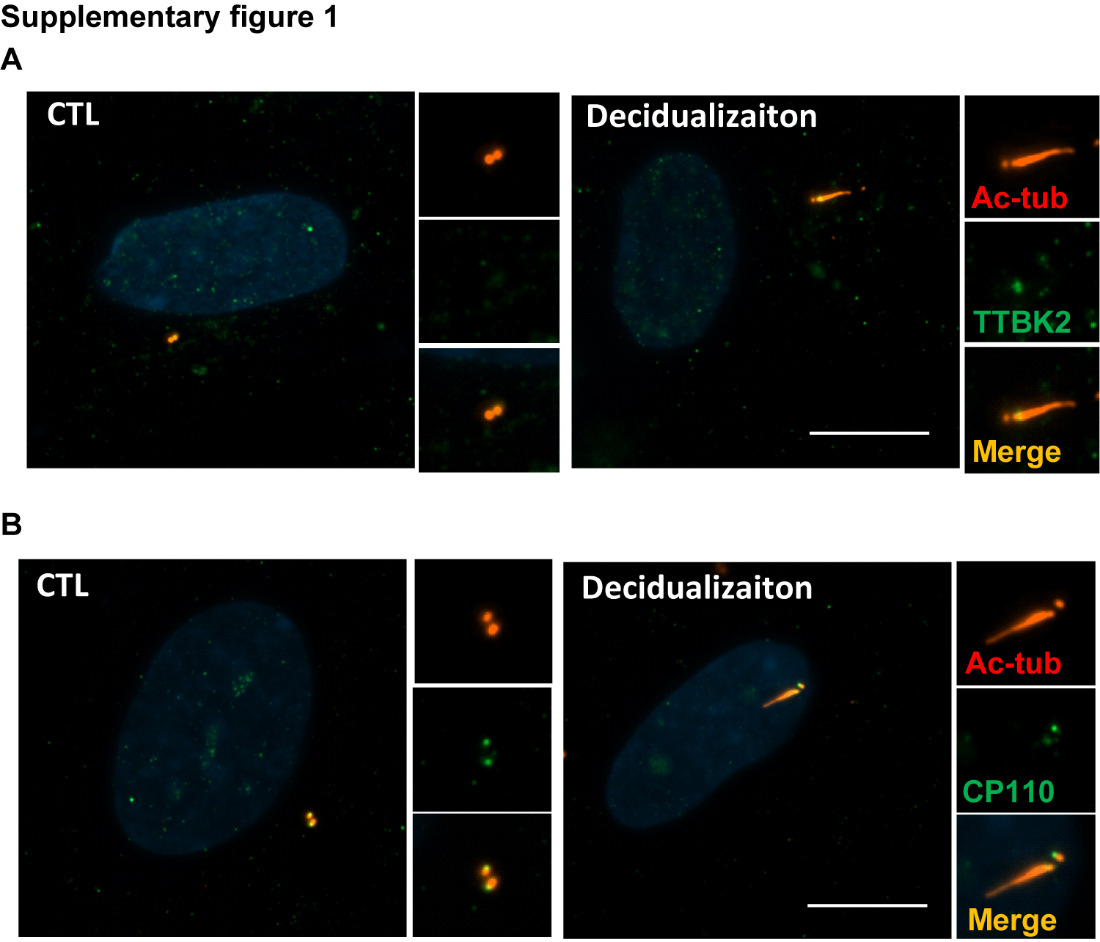


Supplementary Figure 1. Decidual stimulation induces primary cilia formation. (A-B) With the stimulation of decidualization, (A) TTBK2 was recruited and (B) CP110 was removed from the primary cilia. These markers were observed by immunofluorescence staining with antibodies against acetylated tubulin (Ac-tub), TTBK2, and CP110. DNA were stained with DAPI (blue). Scale bar, 10 μm. CTL: control, without decidualization.

Supplementary Figure 2.


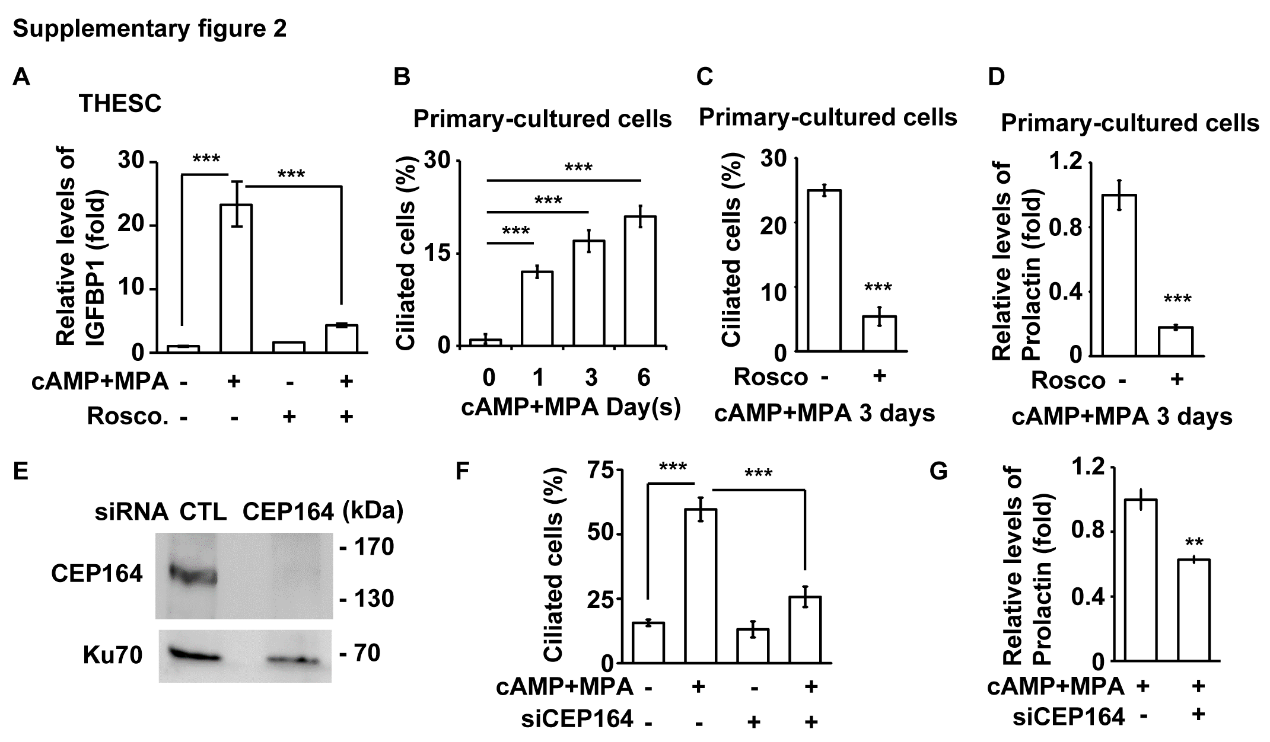


Supplementary Figure 2. Disruption of primary cilia inhibits decidualization.

(A) Treatment of roscovitine reduced decidualization. Quantitative result of the decidual marker IGFBP1. (B-D) Co-treatment of cAMP and MPA induced primary cilia formation in primary-culture cells, and depletion of primary cilia reduced decidualization. (B) Quantitative result of the proportion of ciliated cells in the presence of cAMP and MPA in a time-dependent manner. (C) Quantitative result of the proportion of ciliated cells in treating cells with or without roscovitine during decidualization. (D) Quantitative result of the decidual marker Prolactin. (E-G) Depletion of ciliary gene CEP164 reduced decidual-induced primary cilia and decidualization. (E) Primary cilia gene CEP164 was successfully depleted. The expression of CEP164 was analyzed by western blotting with antibodies against CEP164 and Ku70. (F) Quantitative result of the proportion of ciliated cells. (G) The expression of decidual marker Prolactin was reduced CEP164-deficient cells under decidualization. **p<0.01, ***p<0.001. These results are mean ± SD from three independent experiments. At least 300 cells were counted in each individual group.

Supplementary Figure 3.


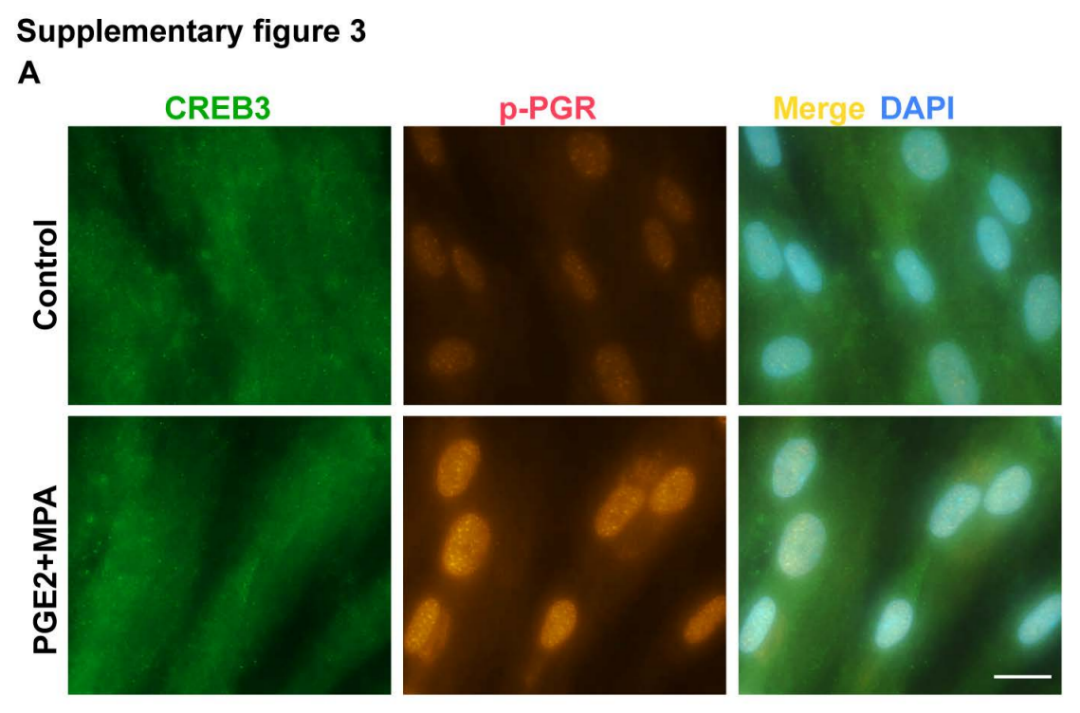


Supplementary Figure 3. CREB3 is not activated during decidualization.

(A) CREB3 was not accumulated in the nucleus upon decidual stimulation. The expression of CREB3 and activated PGR (p-PGR) were analyzed by immunofluorescence staining with antibodies against CREB3 (green) and p-PGR (red). DNA were stained with DAPI (blue). Scale bar, 20μm.

Supplementary Figure 4.


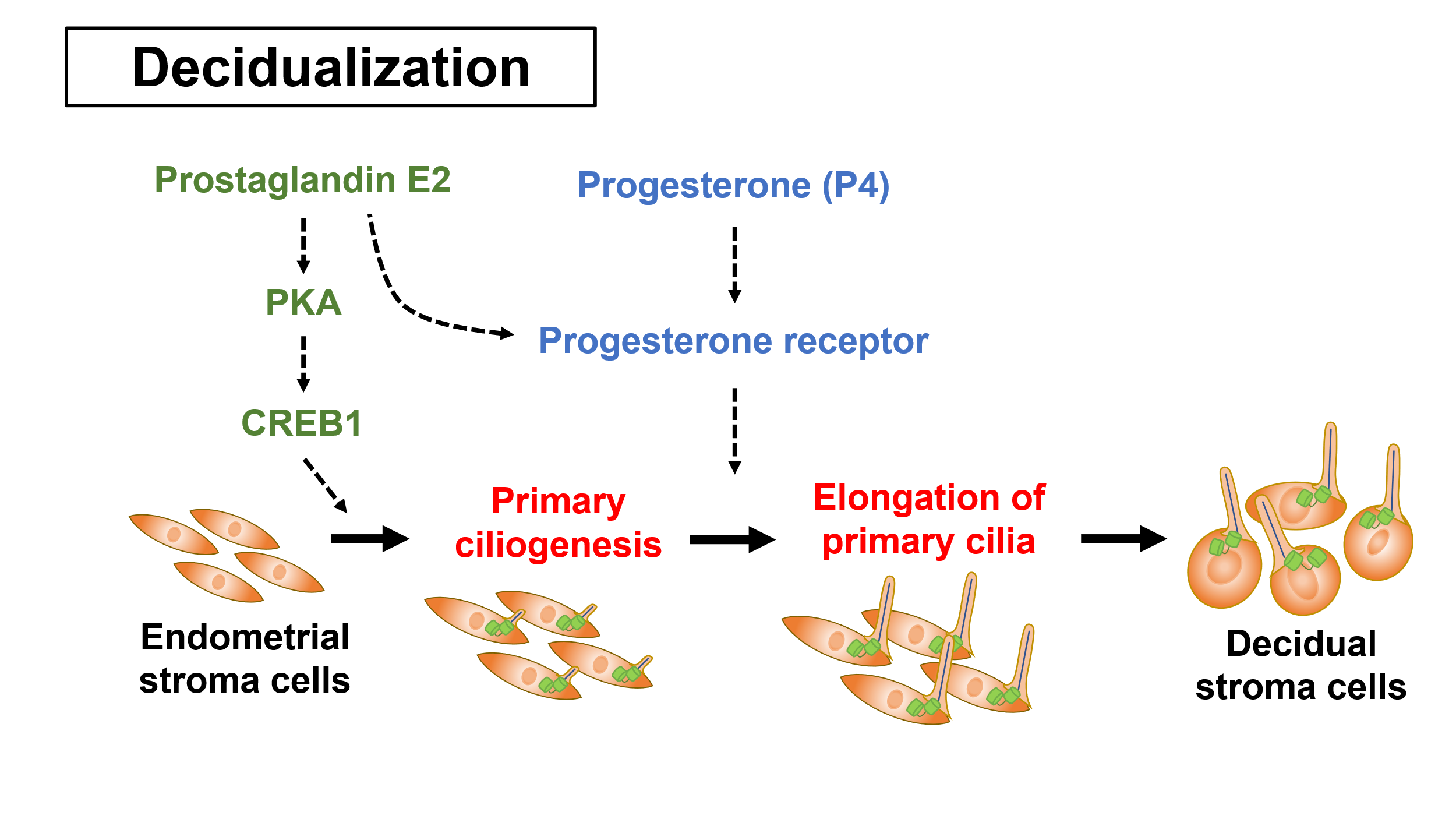


Supplementary Figure 4. Working model of this study. During decidualization, the PGE2-PKA-CREB1 axis induces primary cilia to grow. In addition, PGE2 also upregulates the expression of PGR. Then, upon the stimulation of P4, PGR is activated and promotes the elongation of primary cilia to sustained decidualization.
